# ‘Third wave’ cognitive therapy versus mentalization-based therapy for major depressive disorder. A protocol for a randomised clinical trial

**DOI:** 10.1186/1471-244X-12-232

**Published:** 2012-12-19

**Authors:** Janus Christian Jakobsen, Christian Gluud, Mickey Kongerslev, Kirsten Aaskov Larsen, Per Sørensen, Per Winkel, Theis Lange, Ulf Søgaard, Erik Simonsen

**Affiliations:** 1 Psychiatric Research Unit, Copenhagen University Hospital and Region Zealand, Roskilde, Denmark; 2 Copenhagen Trial Unit, Centre for Clinical Intervention Research, Copenhagen University Hospital, Department 3344, Rigshospitalet, Copenhagen, Denmark; 3 Psychiatric clinic, Psychiatry, Roskilde, Denmark; 4 Psychiatric clinic, Psychiatry, Bispebjerg, Denmark; 5 Department of Public Health, University of Copenhagen, Copenhagen, Denmark

## Abstract

**Background:**

Most interventions for depression have shown small or no effects. ‘Third wave‘ cognitive therapy and mentalization-based therapy have both gained some ground as treatments of psychological problems. No randomised trial has compared the effects of these two interventions for patients with major depression.

**Methods/ design:**

We plan a randomised, parallel group, assessor-blinded superiority clinical trial. During two years we will include 84 consecutive adult participants diagnosed with major depressive disorder. The participants will be randomised to either ‘third wave‘ cognitive therapy versus mentalization-based therapy. The primary outcome will be the Hamilton Rating Scale for Depression at cessation of treatment at 18 weeks. Secondary outcomes will be the proportion of patients with remission, Symptom Checklist 90 Revised, Beck’s Depression Inventory, and The World Health Organisation-Five Well-being Index 1999.

**Discussion:**

Interventions for depression have until now shown relatively small effects. Our trial results will provide knowledge about the effects of two modern psychotherapeutic interventions.

**Trial registration:**

ClinicalTrials: NCT01070134

## Background

### Depression

According to the WHO, major depressive disorder is the second largest healthcare problem worldwide in terms of disability caused by illness [1]. It afflicts an estimated 17% of individuals during their lifetimes at tremendous cost to the individual and society [2,3]. Roughly a third of all depressive disorders take a chronic course [4,5]. Approximately 15% of depressive patients will commit suicide over a 10 to 20 year period [6].

### Antidepressants

Antidepressant medication remains the mainstay in the treatment of depression [7]. However, meta-analyses have shown that most antidepressants presumably only obtain a beneficial effect in severely depressed patients, and even this effect seems to be clinically small [8,9]. As the therapeutic benefits of antidepressants seem to be limited there is an urgent need to identify effective interventions for depression.

### ‘Third wave’ cognitive therapy

Cognitive therapy is one of the most well-known and used psychotherapeutic techniques. Aaron T. Beck originally developed cognitive therapy for depression [10]. Beck believed that critical life events could accentuate hidden negative beliefs, which could generate negative automatic thoughts [10]. These negative thoughts could lead to symptoms of depression, which then could reinforce more negative automatic thoughts [10]. The main goal of the original ‘cognitive model of depression’ is to correct these negative beliefs and thoughts in order to treat the depressive symptoms [10]. One review has questioned if this focus on changing thoughts is an effective element of the cognitive therapy [11]. Our systematic review of randomised clinical trials has shown that classic cognitive therapy versus ‘no intervention’ might be an effective intervention for major depressive disorder. However, the effects of cognitive therapy seem relatively small [12].

During the last two decades modern forms of cognitive therapy have been developed. These techniques are often classified as ‘third wave’ cognitive therapies, including dialectical behaviour therapy (DBT), acceptance and commitment therapy (ACT), schema therapy, mindfulness-based cognitive therapy (MBCT), and metacognitive therapy (MCT) [13]. These therapies have drawn great attention throughout the world, and especially mindfulness has been implemented in numerous different contexts in recent years [14-16]. One systematic review has found that ‘third wave’ cognitive therapy might prevent relapse of depression [17], and preliminary trials indicate that ‘third wave’ cognitive therapy versus ‘no intervention’ or ‘treatment as usual’ is effective for acutely depressed patients [18,19]. ‘Third wave’ cognitive therapies might also be effective interventions for other psychological problems [13,15,20,21]. One trial has shown comparable effects between cognitive therapy and ‘third wave cognitive therapy in non-melancholic depression, but the trial only included 45 participants [22]. Even though evidence is lacking, it seems theoretically possible that the treatment elements of ‘third wave’ cognitive therapy might be more effective than classic cognitive therapy for depressed patients [23]. We have chosen ‘third wave’ cognitive therapy as our experimental intervention because it is practically feasible for us to conduct a trial with this intervention.

### Mentalization-based therapy

In some health-care systems psychodynamic therapy is the most commonly used form of psychotherapy [24]. Mentalization-based therapy is a psychodynamic treatment rooted in attachment and personality theory [25]. It aims to strengthen patients’ capacity to understand their own and others’ mental states in attachment contexts in order to address their difficulties with affect, impulse regulation, and interpersonal functioning [25,26]. Mentalization-based therapy was originally developed to treat borderline personality disorder [27] but is now used to treat a variety of different psychiatric disorders such as depression, eating disorders, substance abuse, and other types of personality disorders [25,26].

Our systematic review of randomised clinical trials has shown that psychodynamic therapy versus ‘no intervention’ might be an effective intervention for major depressive disorder [28], but the effects of mentalization-based therapy versus ‘no intervention’ for major depressive disorder has not been examined in randomised trials [28]. We have chosen mentalization-based therapy as our control intervention because it is practically feasible for us to conduct a trial with this intervention.

### ‘Third wave’ cognitive therapy versus mentalization-based therapy

No randomised clinical trials or systematic reviews have been conducted examining the effects of ‘third wave’ cognitive therapy versus mentalization-based therapy [29].

## Design & methods

We will randomise 84 consecutive patients with major depressive disorder 1:1 to one of two interventions:

1. ‘Third wave’ cognitive therapy, approximately n=42.

2. Mentalization-based therapy, approximately n=42.

The inclusion phase is expected to run for about two years.

### Inclusion

Patients will be referred from general practitioners, practicing specialist physicians, medical departments, and psychiatric departments. Referrals may be denied, either by virtue of the referral papers or via a physician interview. All trial participants will be on sick leave due to psychological illness. To participate in the trial the participant must meet all of the inclusion criteria and none of the exclusion criteria. No special announcement of the project will be made to referrers, so the participants will be similar to patients normally referred to a psychiatric outpatient clinic.

The referrals will be discussed at the patient conference, and relevant patients will be offered a preliminary consultation (collection of master data, which can be used by the therapists during the course of treatment) and a subsequent medical consultation. During the medical consultation relevant patients will be informed of and offered participation in the trial. If the patient agrees to participate in the trial, an appointment will be made for completion of relevant questionnaires. If the patient is included according to the inclusion and exclusion criteria, he or she will be randomised to either ‘third wave’ cognitive therapy versus mentalization-based therapy.

### Inclusion criteria

1. Age from 18 to 65 years.

2. Major depressive disorder (SCID I) [30].

3. Beck’s Depression Inventory (BDI II) > 13 [31].

4. Written informed consent.

### Exclusion criteria

1. Current psychosis, diagnosis of schizophrenia, or schizotypal personality disorder (DSM IV-TR) [32].

2. A significant alcohol or substance abuse (assessed during the preliminary consultations).

3. Initiated or changed medical anti-depressive treatment less than six weeks before randomisation.

4. Pregnancy.

5. No written informed consent.

We have chosen not to include participants over 65 years. This is due to the risk of dementia, which may affect treatment effects. We have chosen BDI > 13 as one of the inclusion criteria to be sure that included participants have significant subjective depressive symptoms. Participants with psychosis, schizophrenia, schizotypal personality disorder, pregnancy, alcohol abuse, and substance abuse are excluded because these groups of participants are traditionally assessed in separate trials.

### Interventions

#### Common to both treatment groups

The intensive treatments will last 18 weeks for each participant. The groups will be ‘slow-open’ (new patients enter the group continually) and will contain a maximum of seven patients. After 18 weeks of intensive treatment every participant will be treated with a follow-up intervention with weekly 1.5-hour sessions of group therapy.

All participants will be offered a communal breakfast twice a week, and group psycho-education for one hour a week. Discussion of treatment plans will be offered after four weeks and after 12 weeks of treatment. The lead clinical consultant, who is not otherwise involved in the trial, will shortly after inclusion offer a consultation regarding psychopharmacological treatment of the psychological problems. The lead clinical consultant will be free to choose any relevant medicine. After the first consultation, medical consultations will be offered by demand of the participant. During the course of treatment, all trial participants with children will be offered participation in a parent support group (four weekly one-hour sessions).

In the absence of one of the therapists (illness, holidays, courses, etc.) the individual therapy will be cancelled. The group therapy will continue, to the extent that this is possible. When participants cancel or do not attend, they will not get compensatory sessions.

### ‘Third wave’ cognitive therapy

Traditional cognitive therapy seeks, in general, to identify irrational thoughts and to ‘correct’ these thoughts [10]. ‘Third wave’ cognitive therapy, and so forth mindfulness (see introduction), is more focused on awareness and acceptance of negative feelings. Our cognitive intervention is primarily based on the principles from mindfulness, so we have chosen to classify this cognitive intervention as ‘third wave’ cognitive therapy [13].

The ‘third wave’ cognitive therapy treatment consists of a weekly individual session (45-50 min.) together with weekly mindfulness-skills training group (1.5 hours).

### The weekly individual session includes

- Introduction of the cognitive model and mindfulness.

- Identification of thoughts, feelings, behaviour, and physical sensations.

- Work on acceptance of feelings and life circumstances.

- Work on assumptions.

- Stress reduction.

- Behavioural experiments and acceptance of difficult feelings.

- Self esteem training.

- Mindfulness training.

- Tools to prevent relapse.

### The weekly mindfulness-skills training group includes

Skills training with teaching of the four basic mindfulness skills ‘FALB’ (training in **F**ocusing, **A**cceptance, **L**abelling techniques, **B**ody awareness) together with self-esteem training and mindful communication. The skills training group will run in a continuous cycle of six sessions. Consequently, participants go through the skills training group’s program three times during the course of the intensive treatment.

For details, please see the manual ‘Third wave’ cognitive therapy - a treatment manual’ for a detailed description of the cognitive intervention [33].

### Mentalization-based therapy

The mentalization-based therapy treatment consists of a weekly individual session (45-50 min.), together with weekly group therapy (1.5 hours).

Mentalising means to understand what lies behind thoughts and activities (e.g., mental states, wishes, needs, goals, and other feelings) [25], and is an active process working both consciously and unconsciously [25]. Mentalization is the process by which we make sense of each other and ourselves. The focus of the mentalization-based therapy is on improving the patient’s ability to mentalise — he or she is trained to mentalise.

### Initial task in mentalization-based therapy

The initial task in mentalization-based therapy is to help the patient to see and learn that her reactions are grounded on something. Feelings and reactions can be understood as a result of what happens in relation to others. Furthermore, the patient is trained in realising that it is not obvious what other people think and feel, and consequently other people cannot know what the patient thinks and feels.

### The five steps in mentalization-based therapy

Mentalization-based therapy has five steps that are repeated a number of times during an intervention period. The *first step* is validation of the patient’s current affect — empathising and supporting the patient. This is followed by clarification of the situation and an elaboration on the patient’s feelings and thoughts; what triggered the feeling; etc. The *fourth step* is ‘basic mentalization’. The patient’s feelings are related to the actual circumstances including other people’s reactions, feelings, wishes etc. The *final step* is to work with interpretation and the transference, including transference interpretations. It is important every step is worked through before going to the next step.

In the first part of an intervention period most time is spend working on step 1-3. Later in an intervention period most time will be spend on step 3-5.

### Therapeutic stance

The therapist must try to demonstrate a ‘mentalising attitude’. The therapist’s attitude must be validating, ‘not-knowing’, and curious questioning the patient about feelings and thoughts. The focus must be on feelings and interpersonal relations. It is of great importance that the therapist identifies when the patient is ‘not-mentalising’, and that the therapist intervenes when this happens. The therapist must assist the patient in regulating the level of the emotions so the patient is able to mentalise; with very strong emotions the patient will not be able to realise what lies behind thoughts and activities, i.e., to mentalise. The therapist must remember not to overestimate the patient’s ability to mentalise, which is a common pitfall. The therapist must help the patient get different perspectives on life events, conflicts, etc.

In mentalization-based therapy the work with the transference process is different from the ordinary psychodynamic way [34]. In mentalization-based therapy it is called ‘mentalising the transference’. ‘Mentalising the transference’ means engaging the patient in reflecting on the relation to the therapist and on the therapist’s mind in the here-and-now. This helps the patient to have ideas of how others se her, by talking about it with the therapist and hearing the therapist’s perspective. In mentalization-based therapy transference is not understood as a repetition of earlier ways of relations, and interpretations based on unconscious processes are not part of the therapy. The counter-transference is managed by the therapists by explicitly telling the patient about the feelings and thoughts the therapeutic process arouse in the therapist. The therapist will always try to present another view and perspective to the patient. In mentalization-based therapy the focus is constantly on ‘mentalising’ interpersonal relations including the relation here-and-now between the patient and the therapist [25,35].

Please see ‘Treatment manual for MBT’ for further details [36].

### Therapists

Each intervention group will have two therapists. The ‘third wave’ cognitive therapy and the mentalization-based therapy therapists will have comparable psychotherapeutic education and experience.

### ‘Third wave’ cognitive therapy therapists

1. The principal clinical investigator (M.D.) is a specialist in general medicine. He has over 10 years of experience in cognitive behavioural therapy and mindfulness and is an approved specialist and supervisor in cognitive behavioural therapy (Danish Psychiatric Association).

2. An occupational therapist with seven years of experience in cognitive behavioural therapy.

### Mentalization-based therapy therapists

1. A mentalization-based therapy licensed psychiatrist with over 20 years of experience with psychodynamic therapy and mentalization-based therapy.

2. A nurse with seven years of experience with psychodynamic therapy and mentalization-based therapy.

### Adherence to treatment manual

All individual sessions will be recorded on an audio recorder and all group sessions will be recorded on video. During the trial an independent research assistant will rate 4 × 10 of the recordings (10 sessions each of: ‘third wave’ cognitive therapy individual therapy, mentalization-based individual therapy, ‘third wave’ cognitive therapy group therapy, and mentalization-based group therapy) to check compliance with the manual. This is done using a checklist and the assessor rates the degree of adherence to the manuals 0-5 (0=no adherence; 5=very high adherence).

### Assessments

Before randomisation: Hamilton Rating Scale for Depression (HDRS) [37], Symptom Checklist 90 Revised (SCL 90-R) [38], Beck’s Depression Inventory (BDI II) [31], World Health Organisation-Five Well-being Index 1999 (WHO 5) [39], Structured Clinical Interviews for DSM-IV Disorders (the depression section of SCID I and SCID II) [30,40], Dimensional Assessment of Personality Pathology-Basic Questionnaire (DAPP-BQ) [41], use of medication, age sex, marital status, and education level.

After 9 weeks of intervention: BDI II WHO 5, use of medication, suicide attempts, and suicides during the intervention period.

After 18 weeks of intervention (end of intensive treatment): HDRS, BDI II, WHO 5, SCL 90-R, use of medication, suicide attempts, and suicides during the intervention period.

After 26 weeks of follow-up treatment: HDRS, SCL 90-R, DAPP-BQ, BDI II, WHO 5, suicide attempts, and suicides during the intervention period.

### Outcome measure hierarchy

#### Primary outcome measure

- HDRS (after 18 weeks of treatment).

#### Secondary outcome measures

- SCL-90-R (GSI- score after 18 weeks of treatment).

- The proportion of patients who achieve remission (after 18 weeks of treatment). We have, pragmatically, chosen to define remission as HDRS below 8 [42].

- BDI II (after 18 weeks of treatment).

- WHO 5 (after 18 weeks of treatment).

#### Other outcome measures

- Adverse events. We will classify adverse events as serious or non-serious. Serious adverse events are defined as any medical occurrence that is life threatening, results in death, or persistent or significant disability, or any medical event, which might jeopardise the patient, or require intervention to prevent it [43]. All other adverse events (that is, any medical occurrence not necessarily having a causal relationship with the treatment, but did, however, cause discontinuation of the treatment) are considered as non-serious.

- Scores on HDRS, BDI, SCL 90-R, WHO 5 after 26 weeks of treatment.

- DAPP-BQ.

### Description of the outcome assessment instruments

#### HDRS

The severity of a depression can be assessed using the Hamilton Rating Scale for Depression (HDRS) [37]. It is the most commonly used assessment scale for depressive symptoms [8,44,45]. It is a continuous, observer-based assessment method and involves 17 items. Using this scale, the degree of depression is assessed (13 to 17 points = mild depression; 18 to 28 points, = moderate depression; over 28 points = severe depression).

#### SCL 90-R

Symptom Checklist 90 Revised (SCL 90-R) [38] is one of the most commonly used instruments for evaluating psychological and psychopathological symptoms. SCL 90-R is a self-reported questionnaire with 90 items. Each item assesses the degree (0-4) of a symptom during the previous week. Global Severity Index (GSI) derived from the questionnaire is designed to measure overall psychological distress.

#### BDI II

Beck’s Depression Inventory (BDI II) [31]. BDI is self-reported questionnaire with 21 items. The BDI score can be interpreted as: 10–18 mild-moderate depression; 19–29 moderate-severe depression; and 30–63 severe depression.

#### WHO 5

World Health Organisation-Five Well-being Index 1999 (WHO 5) [39] is a self-reported questionnaire that attempts to assess the trial participant’s satisfaction and ‘quality of life’. The questionnaire consists of five items and results in a score between 0 and 100.

#### DAPP-BQ

Personality disorder traits can assessed using the Dimensional Assessment of Personality Pathology-Basic Questionnaire (DAPP-BQ) [41]. It is a self-reported questionnaire involving 290 items and 18 scales.

#### SCID

The Structured Clinical Interviews for DSM-IV Disorders (SCID) is an observer-based diagnostic interview used to determine DSM-IV Axis I disorders (major mental disorders, SCID 1) and Axis II disorders (personality disorders, SCID 2) [30,40].

### Reliability tests for assessment

Two experienced psychologists will perform the Hamilton interviews. During the trial period about 20 of the Hamilton interviews will be rated by both interviewers and the correlation will be presented.

The two members of staff who perform the SCID II interviews will rate five SCID interviews before the trial period begins, and the correlation will be presented.

### Data-management

All data will be stored in the principal investigator’s office and at the Copenhagen Trial Unit. All data will be anonymised on conclusion of the project, i.e., after publication of all results. Privacy of trial participants are protected in accordance with the Act on Processing of Personal Data and the Health Act. The project has been notified to the Danish Data Protection Agency.

All completed HDRS interviews, BDI II, and SCL 90 are sent directly to the Copenhagen Trial Unit for data-management.

### Randomisation

The randomisation will be performed by the Copenhagen Trial Unit. A computer will generate a non-stratified randomisation sequence that will be unknown to the investigators. The randomisation sequence will be generated using a block size unknown to the investigators. A research assistant will randomise by calling the Copenhagen Trial Unit providing a personal pin code and patient number.

### Blinding

The Hamilton psychologist interviewer will be blinded to the treatment allocation and will be instructed to avoid questions beside the Hamilton interview. All interviewees will be instructed not to mention which treatment they have been allocated to. Therefore, the HDRS has been chosen as the primary outcome. It is not possible to blind neither the therapists nor the participants to treatment allocation. A blinded statistician at The Copenhagen Trial Unit will perform statistical analyses with the two intervention groups coded as ‘A’ and ‘B’.

The lead clinical consultant performing the medical consultations will not be blinded to treatment allocation.

All information given to the participants before filling in every questionnaire will be the same for every participant and will be standardised.

### Sample size

With a ‘minimal relevant mean difference’ (MIREDIF) between the two compared interventions of 5 HDRS points, an alpha of 0.05 (type I error), a power of 0.90 (type II error of 10%), and a standard deviation (SD) = 7, the sample size calculation shows that a total of 84 participants are necessary. The calculation is performed using the program “Power and sample size calculations”, version 2.1.31.

### Statistical analyses

The primary analyses will be intention-to-treat (ITT) analyses. Per protocol analyses may also be considered.

Continuous outcomes will be compared between the two intervention groups using the univariate general linear model. If the model assumptions cannot be fulfilled with reasonable approximation a non-parametric test will be used (Mann Whitney). The analysis will be repeated using the baseline variable as a co-variate and the results will be discussed in case of major discrepancies between the two results. Binary outcomes will be compared between the groups using logistic regression. The outcomes will be divided into two classes comprising the primary outcome and all the secondary ones respectively. Dmitrienko et al’s gatekeeping testing procedure with parallel inferences will be used to control the family-wise error rate [46]. All raw P values of all outcome comparisons between the two groups will be presented.

As explorative analyses, all of the above analyses will be repeated with the indicator of comorbid personality disorder as well as the interaction between this indicator and the intervention indicator included. If P of the interaction is ≤ 0.05, an analysis of the corresponding subgroups of patients will be conducted.

If more than 5% of the primary outcome measure is missing, multiple imputation will be used (SPSS version 18 or later). If so, the imputation result will be considered the primary result. This analysis will be supplemented by the following sensitivity analysis. Let A be the group where a beneficial significant effect was observed and B the other group. Missing values in group A will be imputed by the mean value of group B and missing values in group B will be imputed by the mean value of group A. The resulting two distributions will probably not be normal and the variances rather small. So the resulting distributions will be compared using a non-parametric test (Mann-Whitney). This test result will be contrasted to that obtained by comparing the two groups without imputed values also using the non-parametric test.

### Prevention of missing values

The primary investigator will ensure that all questionnaires will be completed and will be present at the location where the participant assessments take place. The primary investigator and a research secretary will independently check if all participants have been rated at the relevant time points.

All participants will, in good time, be invited in written letter to participate in the assessments. If the trial participants do not show up or cancel, they will be invited to another assessment. In such circumstances the trial participants will be invited at least three times to a given assessment, and will be contacted on phone or by mail. A research secretary (Anita Jensen) will try to solve any problem that hinders the participant in showing up to the assessment.

### Ethical considerations and regulatory approval

There are no immediate ethical problems regarding this trial. Research has not identified any significant adverse effects or risks from either of the compared interventions — and we do not know which is the best intervention. During the trial period any adverse event will be reported. The trial has obtained approval by the Regional Ethics Committee of Zealand (no: SJ-43), and is registered at the Danish Data Protection Agency (no: 2008-58-0020).

Participants will be informed of the trial in writing and verbally, and written informed consent will be obtained from every participant before inclusion. All trial participants may, on request, be permitted access to further information about the project. The individual therapists and the principal investigator will perform this function. No expense allowance is offered to trial participants.

## Discussion

Our protocol has a number of strengths. First, a trial protocol was registered before randomisation began (ClinicalTrials.gov; no.: NCT01070134). In this protocol the outcome hierarchy and analyses plans were presented and these are published in this present publication. Secondly, the trial protocol has been developed according to good clinical research practice enabling the randomised trial to be conducted with low risk of bias and a high degree of external validity [47-50]. Thirdly, the participants in this trial will be similar to patients normally referred to a psychiatric outpatient clinic, and clinicians can therefore more easily relate the results from this trial to a given clinical context. Fourthly, both interventions will be conducted using manuals and adherence to the treatment manuals will be tested. This makes it possible to implement the interventions in clinical practice, and the two interventions can be assessed in future trials. Moreover, we have used the most commonly used outcome measure in trials assessing the effects of psychotherapeutic interventions for depression (HDRS) [28,37,44,51], as well as other clinically relevant outcome measures (e.g., quality of life, suicide attempts, suicides). The two treatments uses about the same resources, so economical considerations seem less important in the choice between the two.

Our trial protocol has a number of limitations. Due to practical circumstances we have planned to include a limited number of trial participants. This will evidently increase the risk of random errors (‘play of chance’). In calculating our sample size we have used a ‘minimal relevant mean difference’ (MIREDIF) of five HDRS points between the two interventions. Former systematic reviews have shown much less differences in effects sizes between compared interventions for depression [44,51,52]. It could be argued that we have set our presumed MIREDIF unrealistically high. However, as experienced Hamilton interviewers we believe that five HDRS points is a reasonable MIREDIF between the two interventions. A smaller mean difference between two interventions will have limited clinical relevance.

We have chosen to classify our control intervention as ‘mentalization-based therapy’. This intervention was, as mentioned, originally designed to treat borderline personality disorder but is now used in a number of different clinical settings symptoms [25,26]. As we except about 80% of the participants will have comorbidity of depression and personality disorder [53], we believe that mentalization-based therapy will be a relevant control intervention. Mentalization-based therapy is a relatively new intervention and we did not identify any relevant treatment manual we could use. We chose to create our own treatment manual, but due to limited resources the mentalization-based manual became relatively short. The content of the control intervention is therefore less strictly defined and this is a further limitation of this trial.

After the initial 18 weeks of intervention, both intervention groups will be offered a follow-up intervention (1 weekly group session). This follow-up intervention will due to limited resources not be manualised and adherence to treatment manual will not be assessed. The results from the follow-up intervention will be difficult to interpret because the form and content of the both follow-up interventions will be unclear. Furthermore, since the participants will be free to leave the trial after the 18 weeks of intensive treatment (before the follow-up treatment begins), it might be unclear if a certain group of patients will be systematically excluded from the analysis and this might cause biased long-term follow-up results. The participants are free to leave the trial at any time point but based on our experience, we expect a relatively large number of participants to be lost to follow up after the 18 weeks of intensive treatment.

Interventions for depression have until now shown relatively small effect sizes. We want to examine two modern psychotherapeutic methods — and we hope that the results from this trial can contribute to new knowledge about effective interventions for depression.

## Competing interests

We have received external funding for the trial from the Health Science Fund, Region Zealand, Denmark (governmental funding). The amount of funding was altogether 38292 EUR (salary for co-workers, tuition fee for the university, costs for interviews etc.). There are no commercial sponsors. The primary investigator will also be a therapist in the ‘third wave’ cognitive therapy treatment and has developed the treatment manual for the ‘third wave’ cognitive therapy programme. The psychiatrist performing the medical consultations during the trial period will not be blinded to the treatment allocation of the participants and is a mentalization-based therapy therapist. Other authors have no competing interests.

## Authors' contributions

JCJ and CG wrote the first draft. MK contributed with expertise regarding test of adherence to the treatment manuals. KAL, PS, US, and ES contributed with psychiatric expertise. TL and PW contributed with statistical expertise. All authors have contributed to and have approved the manuscript.

## Pre-publication history

The pre-publication history for this paper can be accessed here:

http://www.biomedcentral.com/1471-244X/12/232/prepub
